# The Relationship between Nightmare Experience and Athletes’ Personality Traits and Anxiety

**DOI:** 10.3390/ijerph191912900

**Published:** 2022-10-08

**Authors:** Youteng Gan, Ruohang Wang, Jiangang Li, Xueyu Wang, Hongying Fan

**Affiliations:** School of Psychology, Beijing Sport University, Beijing 100084, China

**Keywords:** negative dreams, NEQ, ZKA-PQ/SF, SAS, stimulating experience

## Abstract

Objective: Frequent nightmare behavior or deep nightmare experiences may harm the physical and mental health and performance of athletes. This study explores the nightmare experiences of athletes, and includes non-athletes with similar experiences for comparison. Methods: The Nightmare Experience Questionnaire (NEQ); Zuckerman–Kuhlman–Aluja Personality Questionnaire, Shortened Form (ZKA-PQ/SF); and Self-Rating Anxiety Scale (SAS) were used. The subjects were 187 athletes (mean age = 20.44 years, SD = 0.85; 91 females, 96 males) and 90 non-athletes (mean age = 20.34 years, SD = 1.65; 52 females, 38 males) who reported having nightmares. Results: A total of 87 athletes (46.5%) reported having nightmare experiences. The athlete nightmare group scored significantly higher in neuroticism than the non-nightmare group, and their anxiety scores were significantly higher than those of non-athletes, who scored higher in aggressiveness, neuroticism, and sensation seeking. Moreover, anxiety, neuroticism, and sensation seeking positively predicted athletes’ nightmare experiences. Conclusions: Personality traits and anxiety levels can be effectively applied to predict athletes’ nightmare experiences.

## 1. Introduction

Dreams are a universal feature of human experience and an epiphenomenon of biology; dream consciousness is thought to be a neurophysiological function performed by the sleeping brain during REM (rapid eye movement) sleep [1]. As one of the typical types of dreams, nightmares are disturbing and well-remembered dreams that are usually accompanied by the sensation of fear, as the theme of nightmares usually involves threats to life or safety, failures, and interpersonal conflicts [2]. Regarding the causes of nightmares, scholars have put forward psychoanalytical and personality models, which emphasize that the regulation or transformation of emotions is the core function of dreams, and the enhancement or destruction of this function is crucial to nightmares [3]. Nightmares often result in waking with rapid orientation and alertness, which not only affects the quality of sleep but also damages the daily function and health of the individual [4]. For example, there are increased negative emotions and self-harming behaviors [5] as well as reduced life satisfaction and work efficiency [6]. A longitudinal study found that individuals with lower levels of psychological well-being were more likely to report negative dreams [7], suggesting that health-related psychological factors may play an important role in causing nightmares. 

Nightmares have been shown to reduce an individual’s tolerance to stress [8]. After exploring the cerebral activity of nightmare disorder patients during the resting state, Shen et al. [9] found that they had the characteristics of increased sensory sensitivity and dysfunction of emotional regulation, so nightmares are also considered a salient target of clinical psychological intervention. Nightmare experiences include the psychological responses to nightmares that dreamers continue to have when they are awake; thus, the frequency of nightmares is a concern. Li et al. [10] found that approximately 5% of people suffer from weekly nightmares, while Wang et al. [11] found that 63.61% of the students they studied reported that they had nightmare experiences. Frequent nightmares are a symptom of underlying psychological distress [12], and nightmare victims with frequent perceptual distortions and nightmares are prone to schizophrenia [13]. In particular, Mack [14] emphasized that nightmares are psychological representations of the early onset of dangerous conditions that are retriggered by life conflicts, major trauma, or both. In brief, further attention needs to be given to the prevalence and harmfulness of nightmares.

For athletes, the stressors of this group mainly included schedule clashes, failure, high training loads, and inflexibility of coaches [15,16]; although athletes are thought to have a stronger ability to resist stress [17], there are different views on the psychological status of athletes. For instance, some researchers believe that the mental health status of athletes is better than that of non-athletes [18,19], but others hold the opposite view [20] or think that there is no difference [21]. Of course, these inconsistencies may be due to different measurement tools and evaluation methods, as well as differences in the study population. Therefore, we believe that comparing the groups of athletes and non-athletes with similar experiences and demographic characteristics may help us to better understand these athletes’ mental health status. In the meantime, as athletes bear more pressure in and out of the arena [22] and need to maintain high levels of competitiveness, these factors will increase their mental health burden to some extent and may induce nightmares. Additionally, some studies have shown a significant correlation between anxiety and nightmare experiences [23,24]. From the perspective of adaptability, Fisher et al. [25] believed that the important function of nightmares is to transfer anxiety to sleep; when dreamers experience fear and anxiety in nightmares, this may ease their corresponding emotions in the real world and help them adapt to the moment. Similarly, anxiety levels can reflect the current stress situation to some extent, and stress increases the frequency of negative emotions in dreams [26], thus increasing the frequency of nightmares. That is, the influence of anxiety on athletes’ nightmare experiences is also worth exploring.

Approximately one-quarter of athletes were identified as having clinically relevant sleep disorders [27]. Turner et al. [28] found that sleep problems were highly prevalent in college student athletes and, interestingly, were also associated with their poorer academic performance. In other words, athletes may be very concerned about their performance in all aspects because of their competitive nature. Hence, sleep disorders as well as nightmares could occur for athletes when they face challenges, which may affect their performance. These sleep and dream disturbances could lead to the formation of a vicious cycle [29], and how to break these cycles and establish effective preventive measures is also a matter of concern. However, there are few studies on athletes’ nightmare experiences.

Frequent nightmares have pathological significance; a meta-analysis showed that both psychological and pharmacological interventions were effective in treating nightmares, where the former seemed to be more durable [30]. Moreover, cognitive behavioral therapy and prazosin are regarded as the main forms of nonpharmacological and pharmacological treatments, respectively [31], but whether they are effective for nightmares with unknown pathological causes remains to be seen. Therefore, we believe that finding economical and effective measures to prevent nightmares has value for athletes, coaches, and sport psychologists.

Personality is a relatively stable characteristic formed by the interaction of genetic, environmental, educational, and other factors [32], and has been successfully applied in studies to predict functional types of personality disorders [33] and anxiety and depression [34,35]. In addition, several studies have found a link between personality traits and nightmare experiences. For example, neuroticism and agreeableness contribute to nightmare distress [36,37]; subjects with high neuroticism and low openness reported that nightmares had more of an impact on them [38], and persons with high conscientiousness reported nightmares slightly more frequently [39]. These studies emphasize how individuals with different personalities react to nightmares, but regrettably, few studies have focused on athletes. Generally speaking, the daily contact environment of athletes is quite different from that of the general population, and the current literature indicates that athletes are more positive [40,41], more conscientious [42], and have specific and unique psychological characteristics [43] when compared with non-athlete samples; these differences may also affect athletes’ behavior, so it is necessary to explore the relationship between personality traits and nightmare experiences in the unique group of athletes.

As noted above, the main purpose of this study was to explore the relationship between athletes’ nightmare experiences and personality traits and anxiety, including non-athletes with nightmare experiences for comparison. Based on the content discussed earlier, our hypotheses were as follows: (1) the personality traits and anxiety levels of athletes are different from those of non-athletes with similar experiences, and (2) personality traits and anxiety levels are associated with athletes’ nightmare experiences.

## 2. Materials and Methods

### 2.1. Participants and Procedures

A total of 277 people, mainly from the Beijing area, participated in this study, including 187 athletes (mean age = 20.44 years, SD = 0.85, ranging from 18 to 23; 91 females, 96 males) and 90 non-athletes who reported having nightmare experiences (mean age = 20.34 years, SD = 1.65, ranging from 18 to 26; 52 females, 38 males). Participants were recruited through personal contacts or coaches/teachers and volunteered to participate in this research survey. The athletes were involved in various sports, with 44 in team sports (e.g., basketball, volleyball) and 143 in individual sports (e.g., gymnastics, swimming); their average training years were 9.12 years (SD = 3.27), and all of them had national-level athlete certificates (second level, first level, or elite level). All participants had received or were receiving undergraduate education.

Participants were required to complete the survey through an internet link and were unaware of the purpose of the study. Informed consent was obtained from all participants before undertaking the study. The study was conducted following the principles of the Declaration of Helsinki and was approved by a local ethics committee (Ethical Approval Document No. 2020156H-1).

### 2.2. Measures

A. Nightmare Experience Questionnaire (NEQ)

The NEQ [44] consisted of 20 items measuring the following four factors: Physical Effect describes the adverse effects of physical health, appetite, and other daily activities after nightmares, such as “I feel that I am getting weak physically because of having nightmares”; Negative Emotion describes the frightened, scared, and helpless emotions during nightmares, such as “I often feel helpless in my nightmares”; Meaning Interpretation describes the dreamer’s attempt to interpret the meaning of the nightmare or to value the information that the nightmare may convey, such as “I can always see the details clearly in my nightmares”; Horrible Stimulation describes the scenes of terrifying activity, adventure, violence, or terrorist events in nightmares, such as “I dreamed about participating in a terrifying activity or adventure”. A 5-point Likert rating scale was used to measure the four factors (1—very unlike me; 2—moderately unlike me; 3—somewhat like and unlike me; 4—moderately like me; and 5—very like me). The NEQ also had an additional item to estimate the frequency of nightmares. The internal alphas for each trait on the NEQ were between 0.64 and 0.83 in the present study.

B. Self-Rating Anxiety Scale (SAS)

The SAS consisted of 20 items and was scored on a 4-point Likert scale (1—none or a little of the time; 2—some of the time; 3—a good part of the time; and 4—most or all of the time). The higher the score, the higher the degree of anxiety. Cronbach’s alpha was 0.80 for the SAS in the present study.

C. The Zuckerman–Kuhlman–Aluja Personality Questionnaire, Shortened Form (ZKA-PQ/SF)

The ZKA-PQ/SF [45] consists of 80 items and measures the following five factors (five items each): aggressiveness (AG), activity (AC), extraversion (EX), neuroticism (NE), and sensation seeking (SS). A 5-point Likert rating scale was used to measure the five factors (1—disagree strongly; 2—disagree somewhat; 3—not well determined; 4—agree somewhat; and 5—agree strongly). The internal alphas for each trait on the ZKA-PQ/SF were between 0.77 and 0.88 in the present study.

### 2.3. Data Analysis

Data analysis was performed using IBM SPSS 26.0 (IBM, Armonk, NY, USA). Chi-squared tests were used to explore associations between sex, sport-related characteristics, and nightmare experiences among athletes. A *t*-test was used to compare the difference in NEQ scales between athletes and non-athletes who reported experiencing nightmares. One-way ANOVAs were used to compare the differences between athletes and nonathletes in the SAS and ZKA-PQ/SF scores. Correlation analyses were used to explore the relationships between NEQ scores, annual nightmare frequency, SDS scores, and ZKA-PQ/SF. Likewise, multiple linear regression (stepwise method) was used to explore the relationships between SAS, ZKA-PQ/SF, and NEQ scales for these athletes and non-athletes, taking the SAS and ZKA-PQ/SF as predictors. A *p* value of <0.05 was considered statistically significant.

## 3. Results

### 3.1. Characteristics of Nightmare Experiences in High-Level Athletes

Among 187 high-level athletes, 87 (prevalence, 46.52%) reported having nightmare experiences, and Chi-squared tests revealed no statistically significant differences between athletes with different sex and sport-related characteristics in their reports of whether they had nightmare experiences (all *p* > 0.05) (see Table 1). Based on the reported results, we divided these athletes into nightmare groups and non-nightmare groups.

Meanwhile, there was no significant difference in sex (χ^2^ = 2.021, *p* = 0.155) or age (*t* = 0.627, *p* = 0.531) between the athlete and non-athlete nightmare groups. In addition, there were also no significant differences in NEQ scores or nightmare frequency between the two nightmare groups (all *p* > 0.05) (see Table 2). 

### 3.2. Anxiety Levels and Personality Traits of Participants

In terms of SAS, the mean anxiety score of the athlete nightmare group was significantly higher than that of the nonathlete nightmare group (*p* < 0.05). In tandem, the mean scores of the nonathlete nightmare group on the three personality factors of aggression, neuroticism and sensation seeking were significantly higher than those of the athlete group (all *p* < 0.05). In addition, the mean score for neuroticism factors was also significantly higher in the athlete nightmare group than in the athlete non-nightmare group (*p* < 0.05). However, there were no significant differences in the mean scores for the activity or extraversion factors between the participants (see Table 3).

### 3.3. Correlations between NEQ, SAS, and ZKA-PQ/SF Scores

In the athlete nightmare group, Pearson’s correlation analysis showed that the physical effect, negative emotion, and NEQ total score were positively correlated with SAS, aggressiveness, and neuroticism, but negatively correlated with extraversion (all *p* < 0.05). Meanwhile, meaning interpretation scores were positively correlated with SAS (r = 0.29, *p* < 0.01), aggressiveness (r = 0.22, *p* < 0.05), and neuroticism (r = 0.46, *p* < 0.01). In addition, horrible stimulation and nightmare frequency were positively correlated with sensation seeking and SAS (all *p* < 0.05), respectively.

Meanwhile, in the non-athlete nightmare group, Pearson’s correlation analysis showed that the physical effect and NEQ total score were positively correlated with SAS and neuroticism (all *p* < 0.05); negative emotion scores were positively correlated with aggressiveness (r = 0.24, *p* < 0.05) and neuroticism (r = 0.30, *p* < 0.01); meaning interpretation scores were positively correlated with activity (r = 0.26, *p* < 0.05) and sensation seeking (r = 0.28, *p* < 0.01); horrible stimulation scores were positively correlated with aggressiveness (r = 0.27, *p* < 0.05), neuroticism (r = 0.25, *p* < 0.05), and sensation seeking (r = 0.31, *p* < 0.01) (see Table 4).

### 3.4. Regression Analysis Results of NEQ by SAS and ZKA/PQ-SF Scales

When considering the prediction of NEQ parameters by SAS and ZKA/PQ-SF scales, the adjusted R^2^ ranged from 0.10 to 0.41 in the athlete nightmare group. Specifically, SAS predicted physical effect (β = 0.45, *p* < 0.01), horrible stimulation (β = 0.26, *p* < 0.05), NEQ total score (β = 0.28, *p* < 0.01), and nightmare frequency (β = 0.33, *p* < 0.01); neuroticism predicted physical effect (β = 0.30, *p* < 0.01), negative emotion (β = 0.47, *p* < 0.01), meaning interpretation (β = 0.48, *p* < 0.01), and NEQ total score (β = 0.40, *p* < 0.01); and sensation seeking predicted meaning interpretation (β = 0.22, *p* < 0.05), horrible stimulation (β = 0.38, *p* < 0.01), and NEQ total score (β = 0.29, *p* < 0.01).

Furthermore, the adjusted R^2^ ranged from 0.07 to 0.29 in the non-athlete nightmare group. Specifically, SAS (β = 0.58, *p* < 0.01) and aggressiveness (β = −0.23, *p* < 0.05) predicted physical effect; neuroticism predicted negative emotion (β = 0.30, *p* < 0.01) and NEQ total score (β = 0.24, *p* < 0.05); and sensation seeking predicted meaning interpretation (β = 0.28, *p* < 0.01), horrible stimulation (β = 0.31, *p* < 0.01), and NEQ total score (β = 0.28, *p* < 0.01) (see Table 5).

## 4. Discussion

The goal of the present study was to investigate the nightmare experiences of athletes. Our research found that in the case of similar demographic characteristics and nightmare experiences, the anxiety level of athletes was higher than that of non-athletes. In addition, non-athletes scored higher in aggressiveness, neuroticism, and sensation seeking, while the nightmare group (athletes) scored higher in neuroticism than the non-nightmare group (athletes). Meanwhile, anxiety and personality traits can predict the nightmare experiences of athletes. To a degree, this study helps to explain the relationship between athletes’ personality traits, anxiety levels, and nightmare experiences.

In terms of nightmare experience, we found that 46.52% of athletes reported this experience, which was similar to the finding of Mello et al. [46] that 39.1% of athletes had nightmares before competitions. Too-frequent nightmares or too-intense nightmare experiences may affect an individual’s mental state; that is, if not handled properly, nearly half of athletes found in this study will be at risk of nightmare disorders, which requires adequate attention. Meanwhile, concerning the score of the NEQ scale and the frequency of nightmares, there was no difference between athletes and non-athletes, which may be because sports-related characteristics were not related to nightmares and the demographic characteristics of the two were similar. In short, both athletes and non-athletes had a similar understanding of and feelings about nightmares, as well as the impact of perceived nightmares on themselves.

It is important to note that when comparing anxiety scores, we found that the athlete nightmare group scored higher than the non-athlete nightmare group. Previous studies on the mental health of athletes and non-athletes have been rich but inconsistent [47,48], so we tried to compare the research groups with similar experiences from another perspective. Based on the research results and combined with the environmental changes and challenges that athletes may encounter during their development, we put forward an explanation. Although athletes’ ability to resist stress and strain is recognized as strong, is this ability limited to these relatively single pressures of competition and training? Meanwhile, as athletes advance in age, they will face pressures such as academic performance and more complex interpersonal relationships that they did not or were rarely worried about before [49]. They may not be as prepared or able to handle these problems compared to non-athletes. Furthermore, Martin [50] found that athletes and non-athletes reported the same level of stress; non-athletes responded to stress by listening to music and socializing with friends and family, while athletes relieved stress mainly through exercise [51], which also indicates that athletes take slightly singular measures in coping styles. Therefore, this may cause athletes to choose to bear stress alone when experiencing nightmares, coupled with their more negative attitude toward seeking help than non-athletes [52], which can exacerbate their anxiety. In brief, it cannot be taken for granted that athletes will be better at psychological endurance when experiencing setbacks, and this result can also deepen our understanding of athletes. 

Regarding the difference in personality trait scores, we found similar results to Pačesová and Šmela [53] in the dimension of aggressiveness; they thought that non-athletes were more aggressive than athletes, and there was no difference in the level of aggression among athletes. To a degree, sports training provides a distraction from negative emotions and tension release, thereby reducing aggression [54]. Hence, whether athletes are confused by nightmares or not, daily training may increase their likelihood of releasing aggression, which is also one of the benefits of sports activities from the perspective of personality building. In tandem, the group with nightmare experiences had significantly higher scores on neuroticism. Showing a high level of neuroticism traits has been shown to be associated with the sensitivity of negative emotional experiences, emotional vulnerability, and increased emotional exhaustion [55]. Our research further shows that the occurrence of nightmare experiences is associated with showing a high level of neuroticism traits in both athletes and non-athletes. However, in terms of sensation seeking, our findings were not consistent with previous studies [56]. Notably, the non-athletes in this study belong to a relatively special group, and studies with similar themes have found that nightmare distress is positively correlated with sensation seeking [57], suggesting that sensation-seeking traits not only affect individuals’ experience of stimulus-related and interest in novelty, but also may affect their nightmare experience. Moreover, similar to aggressiveness, athletes may experience more stimulation during training or competition, which can satisfy their pursuit of that feeling to some extent. Thus, it can be considered that sensation seeking may have less impact on their nightmares than on those of non-athletes. Consequently, a full understanding of the training and living environment of athletes might help us to understand the role of these factors in the shaping of personalities. As expected, athletes show unique personality characteristics when compared with non-athletes who have had similar experiences.

Our results also reveal a correlation between athletes’ anxiety, personality traits, and nightmare experiences. Specifically, anxiety level was positively correlated with the physical influence dimension of the NEQ, indicating that the anxiety of individuals was proportional to the impact of nightmares on their quality of life. Anxiety has been proven to impair the quality of life of individuals [58], and we believe that this impairment process also includes the effects of nightmares. In addition, the positive correlation between anxiety and negative emotions, meaning interpretation, and the total score of the NEQ further emphasize the strong association between negative emotions and nightmares. Additionally, as consistent with the results of Nguyen et al. [23], we found that anxiety and nightmare frequency also have a significant relationship among athletes. Likewise, our results suggest that, in terms of personality, aggressiveness and neuroticism have a similar positive correlation with nightmare experience, but the correlation of neuroticism is significantly stronger. For aggressiveness traits, studies have found that the dreams of patients with abnormal sleep behavior contain a higher proportion of aggressive content [59], suggesting that nightmares may have a mutually reinforcing relationship with aggression. Meanwhile, the connection between the neuroticism traits that represent emotional stability and the nightmare experience as a negative event is self-evident. In addition, a study on the dream recall frequency of college students (n = 183) showed that extraversion was negatively correlated with nightmare recall frequency [60]; our study also supports the notion that extraversion has the same relationship with the total score of the NEQ. Furthermore, individuals with higher scores for horrible stimulation will experience more horror plots in nightmares, which is similar to the pursuit of stimulation by sensation seekers, so it is reasonable that there was a positive correlation between the two. Overall, the results help us understand the relationship between the depth of athletes’ nightmare experiences and emotional status, as well as personality traits.

Our analyses examining the predictive effects of anxiety level and personality traits on nightmare experiences and frequency of nightmares for athletes reveal that anxiety and neuroticism positively predict physical effects. Neuroticism has been found to be a strong association and predictor of many different mental and physical disorders [61] and is similar to anxiety, both of which have an impact on quality of life [62,63]. In other words, the higher the anxiety and neuroticism of athletes, the greater they feel that their lives are disturbed by nightmares, and these characteristics and dreams may fall into a vicious cycle. Thus, it is necessary to guide athletes to correctly recognize and deal with nightmares. Meanwhile, neuroticism and sensation seeking predict meaning interpretation, indicating that individuals who show high traits in these areas tend to believe that nightmares contain important information that they value. It is worth mentioning that in their study, Köthe and Pietrowsky [38] found that subjects who were highly neurotic, stressed, or dissatisfied with their lives tended to dwell on their nightmares, so they often felt uneasy and tried to explain their dreams. Furthermore, as far as terrorist stimulation is concerned, positive predictive effects of anxiety and sensation seeking were found. To a degree, negative effect intensity can significantly predict fear of emotions [64], while sensation seekers perceived the world as less dangerous [65] and still reported that they were willing to take more risks while driving even after watching a scary video about the consequences of dangerous driving [66]. This pursuit of excitement may also be reflected in their nightmare experiences. It is worth noting that athletes’ anxiety levels also predicted the frequency of nightmares, underscoring the importance of helping them to master ways to deal with negative emotions. In terms of total scores, our research shows that neuroticism, sensation seeking, and anxiety play a role in predicting the overall level of athletes’ nightmare experiences, which also suggests that sports workers should pay more attention to the behavior of these athletes to prevent their nightmare experiences from continuing and developing into mental disorders.

Notably, we also found neuroticism and sensation seeking to be predictors of overall nightmare experiences in non-athletes. That is, physical health concerns and emotional complaints after nightmares may be driven primarily by personality factors, both for athletes and non-athletes. Undoubtedly, the combination of neuroticism and sensation seeking plays an important role in determining the depth of nightmare experiences; neuroticism, in particular, plays a key role in whether individuals report more intense nightmare experiences. Hence, our research confirms that personality traits are associated with nightmare experiences, which is one of the factors that Wang et al. [67] hope to verify in future studies.

Notwithstanding, the present study suffers from some limitations. First, our measurements of the nightmare frequency of the participants were based on retrospective memories, which may have some impact on the results of the study. Future studies can consider recording participants’ recent sleep quality in detail to measure their nightmare frequency more accurately. Second, we did not measure the participants’ stress levels or negative life events, which may be associated with nightmare experiences. In future studies, researchers could try to include these variables for further analysis. Finally, the study was a cross-sectional study that could only emphasize the correlation of study variables, but could not reveal causation between them. In future studies, researchers should implement longitudinal or experimental designs to deepen the understanding of the issues raised in the current study.

## 5. Conclusions

In light of the above, this study has shown that compared to non-athletes with similar demographic characteristics and nightmare experiences, athletes have higher levels of anxiety and worse emotional stability. Meanwhile, athletes with high neuroticism, high sensation seeking, and high anxiety may have more intense nightmare experiences and more frequent nightmares under the influence of anxiety. Therefore, pre-assessment of personality traits and regular measurement of anxiety levels can help us effectively identify athletes who are likely to be troubled by nightmares, and timely appropriate interventions can be taken to reduce the risk of nightmare disorders and minimize negative impacts. Our study confirms the influence of personality traits and anxiety levels on athletes’ nightmare experiences, which further provides ideas for the prevention and treatment of nightmares and has certain application significance for sport psychologists and coaches.

## Figures and Tables

**Table 1:** Comparison of nightmare experiences among athletes with different sex and sports-related characteristics (n = 187).

Variables	Category	No. (%)	No. Experiencing Nightmares (%)	χ^2^	*p*
Sex	Male	96 (51.34%)	41 (42.71%)	1.154	0.283
	Female	91 (48.66%)	46 (50.55%)		
Type of sport	Team sports	44 (23.53%)	19 (43.18%)	0.258	0.611
	Individual sports	143 (76.47%)	68 (47.55%)		
Skill level	Second level	85 (45.45%)	40 (47.06%)	0.946	0.623
	First level	78 (41.71%)	38 (48.72%)		
	Elite level	24 (12.83%)	9 (37.50%)		
Training years	≤9	97 (51.87%)	46 (47.42%)	0.065	0.798
	>9	90 (48.13%)	41 (45.55%)		
Total		187 (100%)	87 (46.52%)		

**Table 2:** Comparison of NEQ dimensions between the athlete nightmare group and the non-athlete nightmare group (mean ± SD).

Dimension	Athlete Nightmare Group (n = 87)	Non-Athlete Nightmare Group (n = 90)	*t*	*p*	Cohen’s d	95% CI	
						LL	UL
NEQ							
Physical Effect	6.99 ± 2.53	6.60 ± 2.47	1.03	0.30	0.16	[−0.35, 1.13]	
Negative Emotion	17.25 ± 5.03	16.26 ± 4.94	1.33	0.19	0.20	[−0.48, 2.48]	
Meaning Interpretation	16.16 ± 4.02	16.01 ± 3.99	0.25	0.80	0.04	[−1.04, 1.34]	
Horrible Stimulation	13.92 ± 4.35	14.26 ± 4.25	−0.52	0.60	0.08	[−1.61, 0.94]	
Total score	54.32 ± 12.22	53.12 ± 11.16	0.68	0.50	0.10	[−2.27, 4.67]	
Nightmare frequency (time/year)	11.89 ± 9.39	13.27 ± 12.79	−0.82	0.42	0.12	[−4.72, 1.96]	

Note: NEQ = Nightmare Experience Questionnaire.

**Table 3:** Descriptive statistics and differential analysis of participants’ scores on the SAS and ZKA-PQ/SF scales.

Scales	Athlete Nightmare Group (n = 87)	Athlete Non-Nightmare Group (n = 100)	Nonathlete Nightmare Group (n = 90)	F	*p*	η^2^
SAS	42.59 ± 9.10	40.40 ± 8.73	39.96 ± 7.29 *	2.49	0.09	0.02
ZKA-PQ/SF						
AG	41.49 ± 11.71	40.08 ± 10.62	46.86 ± 10.25 **	10.04	<0.001	0.07
AC	48.13 ± 9.33	46.78 ± 11.63	47.52 ± 7.32	0.46	0.64	0.003
EX	54.74 ± 11.33	53.44 ± 10.86	55.78 ± 8.98	1.20	0.30	0.01
NE	42.71 ± 12.51	39.30 ± 11.06 *	46.96 ± 8.84 *	11.71	<0.001	0.08
SS	47.52 ± 9.53	45.61 ± 8.99	51.28 ± 8.76 **	9.42	<0.001	0.06

Note: * *p* < 0.05; ** *p* < 0.01 vs. athlete nightmare group. SAS—Self-Rating Anxiety Scale; ZKA-PQ/SF—Zuckerman–Kuhlman–Aluja Personality Questionnaire, Shortened Form; AG—aggressiveness; AC—activity; EX—extraversion; NE—neuroticism; SS—sensation seeking.

**Table 4:** Pearson correlation matrix of NEQ, SAS, and ZKA-PQ/SF of the athlete nightmare group (n = 87) and the non-athlete nightmare group (n = 90).

	SAS	AG	AC	EX	NE	SS
Athlete nightmare group						
NEQ						
Physical Effect	0.60 **	0.40 **	0.03	−0.45 **	0.53 **	−0.12
Negative Emotion	0.32 **	0.34 **	0.06	−0.31 **	0.47 **	0.12
Meaning Interpretation	0.29 **	0.22 *	0.12	−0.11	0.46 **	0.18
Horrible Stimulation	0.18	0.20	0.19	0.03	0.16	0.32 **
NEQ Total Score	0.41 **	0.37 **	0.14	−0.24 *	0.51 **	0.20
Nightmare Frequency (time/year)	0.33 **	0.15	0.01	−0.21	0.13	0.07
Non-athlete nightmare group						
NEQ						
Physical Effect	0.51 **	−0.05	0.04	0.10	0.26 *	−0.04
Negative Emotion	0.07	0.24 *	0.02	0.08	0.30 **	0.16
Meaning Interpretation	0.13	0.17	0.26 *	0.17	0.03	0.28 **
Horrible Stimulation	0.11	0.27 *	0.25 *	0.05	0.10	0.31 **
NEQ Total Score	0.23 *	0.26 *	0.21	0.09	0.24 *	0.28 **
Nightmare Frequency (time/year)	0.14	0.01	0.05	0.05	0.02	0.07

Note: * *p* < 0.05, ** *p* < 0.01. NEQ—Nightmare Experience Questionnaire; SAS—Self-Rating Anxiety Scale; ZKA-PQ/SF—Zuckerman–Kuhlman–Aluja Personality Questionnaire, Shortened Form; AG—aggressiveness; AC—activity; EX—extraversion; NE—neuroticism; SS—sensation seeking.

**Table 5:** Stepwise multiple linear regression for predicting NEQ from SAS and ZKA/PQ-SF scales in athlete nightmare group (n = 87) and non-athlete nightmare group (n = 90).

	Athlete Nightmare Group		Non-Athlete Nightmare Group	
	Adjusted R^2^	β (B, Standard Error) Predictors	Adjusted R^2^	β (B, Standard Error) Predictors
NEQ				
Physical Effect	0.41	0.45 (0.13, 0.03) SAS **	0.29	0.58 (0.20, 0.03) SAS **
		0.30 (0.06, 0.020) Neuroticism **		−0.23 (−0.05, 0.02) Aggressiveness *
Negative Emotion	0.21	0.47 (0.19, 0.04) Neuroticism **	0.08	0.30 (0.17, 0.06) Neuroticism **
Meaning Interpretation	0.24	0.48 (0.15, 0.03) Neuroticism **	0.07	0.28 (0.13, 0.05) Sensation Seeking **
		0.22 (0.09, 0.04) Sensation Seeking *		
Horrible Stimulation	0.15	0.38 (0.17, 0.05) Sensation Seeking **	0.09	0.31 (0.15, 0.05) Sensation Seeking **
		0.26 (0.12, 0.05) SAS *		
Total Score	0.35	0.40 (0.39, 0.10) Neuroticism **	0.12	0.28 (0.36, 0.13) Sensation Seeking **
		0.29 (0.37, 0.11) Sensation Seeking **		0.24 (0.30, 0.13) Neuroticism *
		0.28 (0.37, 0.14) SAS **		
Nightmare Frequency (time/year)	0.10	0.33 (0.34, 0.11) SAS **	—	— —

Note: * *p* < 0.05, ** *p* < 0.01; B values are unstandardized coefficients. NEQ—Nightmare Experience Questionnaire; SAS—Self-Rating Anxiety Scale; ZKA-PQ/SF—Zuckerman–Kuhlman–Aluja Personality Questionnaire, Shortened Form.

## Data Availability

The datasets of the current study are available from the corresponding author upon reasonable request.
